# Risk Perception of Food Safety by School Food-handlers

**Published:** 2014-03

**Authors:** Maria Grossi Machado, Estelamaris Tronco Monego, Maria Raquel Hidalgo Campos

**Affiliations:** ^1^Postgraduate student of Program in Nutrition and Health, Nutrition School, Federal University of Goiás, Brazil;; ^2^Professor of Nutrition School, Federal University of Goiás, Brazil

**Keywords:** Food, Perception, Risk, School meal, Brazil

## Abstract

An exploratory descriptive study was conducted with a qualitative approach that used focus groups. The objective of this study was to identify the risk perception of food safety by school food-handlers. The results indicated that the food production process has certain inadequacies, including the weak risk perception by the food-handlers regarding the student's health. The students, the pedagogical team, and the principal contribute to this behaviour, which can affect the quality of the final product—the served meal. The social devaluation of the food-handlers is also discussed. It is necessary to improve the food-handlers’ training sessions, with the purpose of modifying risk perception and to allow the school community to be involved in healthy and safe feeding practices.

## INTRODUCTION

In Brazil, the National Policy for Feeding and Nutrition (PNAN) promotes the Feeding and Nutritional Safety (SAN) and the Human Right to Proper Feeding (DHAA) policies. The principles and guidelines of PNAN describe strategic actions for feeding and nutrition and highlight the importance of the National Program of School Feeding (PNAE), which is the model for the implementation of school feeding programmes in different countries (1).

Interministerial Administration Rule # 1010 from the Health and Education Ministries (MS/MEC) and RDC Resolution # 216 from the National Health Surveillance Agency (ANVISA) are examples of the shared responsibilities between the healthcare and the educational programmes on the implementation of good handling practices during the production and distribution of foods in schools (2,3).

Food production in schools is done by food-handlers who, throughout PNAE history, have acquired substantial knowledge due to increasing complexity not only in the handling of premade meals but also in the preparation of regional and fresh foods (4).

According to Costa, Lima, and Ribeiro, the work of food-handlers is socially devalued. Food-handlers are composed of mestizo and black women of low educational background, who live in poor financial and social conditions. Their work conditions are characterized by low wages, high workload due to insufficient staffing, and increasing responsibilities, including the hygienic handling and preparation of foods (5).

The school community, including the food-handlers, the pedagogical team, the principal, and the parents, need to be trained in the SAN principles so that these principles become a model for the production of innocuous and healthful foods, based on the guidelines described in the Global Strategy (OMS) and the Codex Alimentarius (6,7).

Certain studies have emphasized the technical aspect of the food-handlers’ training sessions rather than the educational process (4,5,8). Thus, the challenge lies with the trainer who must be capable of thinking about the process beyond the typical transfer of knowledge involved in standard technical training (9).

The assessment of the food safety risk in school meals, i.e. the probability of an adverse event or threat against human health and the perception of this risk can be the starting points that would allow changes in the food-handlers’ knowledge, skills, and attitudes (10).

The risk perception is based on internal and external factors, such as experience, beliefs, and images; the perceived behaviours rely on resources other than the individual's scientific knowledge (11). Studies suggest that this risk perception is based on experience, information, and cultural background; however, these components are not limited to population group factors (12,13).

There have been a limited number of qualitative studies that have assessed the risk perception of food safety by school food-handlers (4,14). For the assessment of this risk perception, it is crucial to include a subjective group of individuals. This study has assessed the risk perception of food safety by school food-handlers and identified factors that hinder the proper hygienic practices within the school environment.

## MATERIALS AND METHODS

This study is an exploratory descriptive study that employed a qualitative approach with the use of a focus group that consisted of food-handlers from public schools.

A focus group study, which is very effective in qualitative research, examines the knowledge and experience of a group of individuals about disease or health-related behaviours. Focus groups allow the formulation of questions about beliefs, attitudes, and perceptions within specific groups of individuals; furthermore, they allow the development of hypotheses for other studies that use this exclusive methodology (15,16).

The focus groups consisted of school food-handlers who, for the purpose of this study, had previously participated in a specific training session. This training had several approaches, including notions on hygiene and food-handling. It was a part of another project titled “Evaluation and monitoring of the food quality offered in school meals” (17). The inclusion criteria were: not being sick or on personal leave, not planning to retire during the study and able to use the public transportation system. Punctuality in the first meeting was also considered an inclusion criterion.

**Figure 1.**
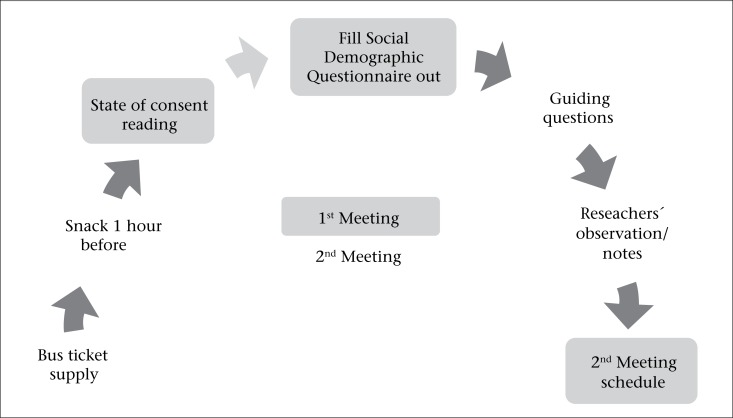
Leading focus group meetings

Two focus group meetings (held in February and March 2010) were recorded and conducted with the same group of food-handlers, and three trained moderators discussed the following topics: risk perception of food safety; knowledge about food hygiene and handling; knowledge about foodborne diseases; training sessions on foodborne diseases; and suggestions for subsequent training sessions. Each meeting lasted one hour and thirty minutes. The way the focus groups were led is shown in Figure 1.

**Figure 2.**
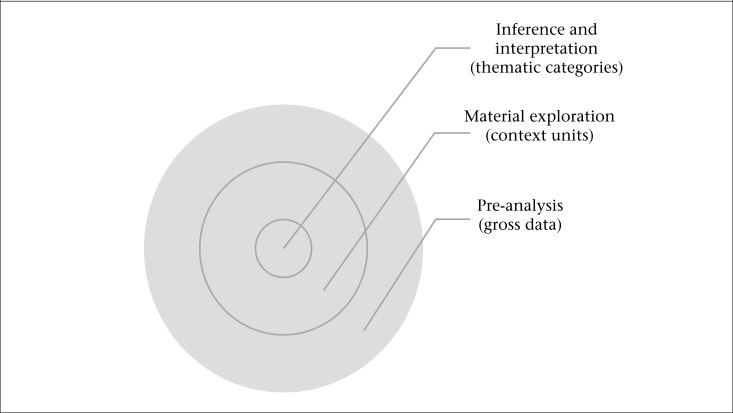
Methodological path to reach the thematic categories and context units

The data were analyzed through Content Analysis consisting of “a set of communication analysis techniques aiming to obtain, by systematic procedures and objectives of messages content description (quantitative or qualitative) indicators that allow the inference of knowledge concerning the production conditions and reception of these messages” (18). The texts from the focus group were transcribed, followed by a transformation of de gross data; the context units were elaborated, and then the thematic categories were analyzed. The methodological path to reach the thematic categories and context units is shown in Figure 2.

The study, which was approved by the Committee of Ethics in Research from the Federal University of Goiás (# 148/2009), met the ethical principles of the Declaration of Helsinki (2008), the World Medical Association, and CNS Resolution 196/96.

## RESULTS

Ten food-handlers (100%) and eight food-handlers (80%) participated in the first and second meeting respectively. Both groups were composed of the same people and two of them gave up on the second meeting. All the food-handlers were female aged 37 to 58 years, who had children and whose source of income was their job in school. One food-handler (10%) did not own a house; all of them had access to public water and sewers services, electricity, refrigerators, stoves, bathrooms, showers, and televisions in their residences.

The thematic category was identified according to the topics of the focus group meeting: risk perception of food safety; knowledge about food hygiene and handling; and knowledge of foodborne diseases.

The thematic categories and the context units are presented in the Table.

### Risk perceptions by food-handlers

This thematic category analyzed the food-handlers’ interpretation of threats to health, based on their beliefs and convictions and not on facts or scientific information (11).

The context units revealed from the answers of the focus groups identified factors relating to the school community with responsibility over the actions pertinent to the food safety risk in school meals.

**Table.** The thematic categories and context units that emerged from the focus group meetings

Thematic category	Context unit
1. Risk perceptions by the food-handlers	Food-handlers’ knowledge
2. Sources of information	The role of schools in food hygiene
3. The food-handler as a protagonist in the teaching-learning process	The role of students in food safety
	The role of pedagogical team in food safety risk

### Food-handlers’ knowledge

This context unit identified what the food-handlers know and what they do to effectively protect the students’ health. Their knowledge regarding food preparation was evident in the food-handlers’ statements:

We are aware of food safety risks everytime we are going to prepare a meal…. because of the presence of bacteria, which increases the risk of foodborne diseases in the student…. You have to be very careful…. When we finished preparing a meal, we store it in the refrigerator right away; what remains is thrown away.

Although they are aware of risky behaviours, such as the use of inadequate temperatures, this awareness does not stop the food-handlers from performing tasks in an incorrect manner:

You remove the meat from the freezer that will be served for lunch because the meat has to thaw fast for the preparation of the pre-lunch. The meat that is left outside represents a risk!

The limitations of the workplace, including the lack of infrastructure and equipment, were also discussed. Food-handlers were aware of proper food-handling procedures; however, in daily work, those procedures were not actually enforced:

The little bowls were not enough for the students; so, we used the dirty ones.

Behaviours that put the health of students at risk were reported by the food-handlers who recognized that those were inappropriate procedures:

It happened with the fruit salad…. We know that it is dangerous but we did not care…. We have a ‘cutter’ for cutting bananas; if the banana falls on the floor and nobody is watching [laugh], we take it and put it inside the bowl.

This study found no association between behaviour and its consequences, most likely because the school environment represents an extension of what is done at home.

If a student asks for a second food or for the leftovers in the cooking pans, he/she is served by the food-handler even though there is a per-capita ration that is specified by the Education Bureau. The group believes that these rations are not adequate:

Each school has its menu and its per-capita ration…For instance, take this small bowl; several students can eat three bowls like this one, and it is still not enough to feed them.

### Role of schools in food hygiene

The focus group reported that most public schools do not have appropriate work conditions in the Meal Producing Unit (UPR). However, they consider that some schools have better infrastructure, including disposable glasses, plates, and routine replacement of the silverware:

Only the glass is disposable in this school. The juice, the smoothies, and the liquids are served in disposable glasses. However, the rice, the chicken soup, the beans, and the toasted manioc flour go in a bowl…. Our coordinator asks us to separate the bowls and the spoons that have defects or scratches, and she will get rid of them.

The food-handlers also discussed some of their overlapping responsibilities, including the cleaning of bathrooms, classrooms, and playgrounds:

Sometimes, you are quickly cleaning the bathroom, the classrooms, and then you are preparing the snacks…So, it involves a variety of work functions like these.

### Role of students in food safety

The focus group pointed out that the students play a role in the food safety risk:

We have found bowls inside the students’ bathrooms.

The risk in food safety is augmented when the student brings spice high in sodium from their homes to add to their school meals since the prevalence of hypertension in children and adolescents had increased every year:

We see this problem a lot: the students bring a seasoning or spice from their homes so that they can add it to their food because they claim it is not seasoned enough.

### Role of pedagogical team in food safety risk

The focus group reported that teachers often have inappropriate attitudes that reinforce the risky behaviours of the students:

The other day, I was wearing gloves, and the snack's tray was covered with a dishcloth…. As I began serving the snack to the student, the teacher took a donut, split it with her hands full of chalk, put the other half back on the tray and said, “I will eat just a little so that I do not get fat.”

In some schools, the employees respect the standards (e.g. walking into the kitchen, wearing a cap); however, the group reported that most of the time the employees (i.e. the principal and the administrative staff) do not follow the hygiene standards.

A hair found in the school may come from the teacher´s head but a hair found in the pan is from the food-handler. So, neither teachers nor the students will have any hair in their meals.

This study suggests that the responsibility of food safety falls on the food-handlers because they work directly in the food production and preparation; however, the other school members do not see themselves as responsible for their actions.

### Sources of information

This thematic category identified the different methods to access information about food sanitation. The results indicated that these methods were unreliable sources.

The focus group indicated that, through different training sessions, they had obtained information about food, personal and environmental hygiene and food contamination:

You put food in the bowls, cover them, and put the bowls in the refrigerator. Then, by heating the food, you are killing any bacteria that might be present.

In these training sessions, the food-handlers also gained knowledge regarding certain food ingredients that may cause disease. The group knew that the use of excessive amounts of sugar, salt, or oil posed a food safety risk:

During the food preparation, we add the right quantity of salt and oil, for the sake of the students’ health; students like consuming fatty foods. You have to think about the adverse effects of excessive amounts of fat on the students’ health.

However, the food-handlers are not aware of certain foods and food preparation methods that have higher risks of contamination, and they often question the proper way of storing leftovers. The information in this area usually comes from different sources (i.e. friends, the radio, newspapers, and supermarkets). The information, though not always correct, was evident during the focus group meeting while discussing the food preparation process:

This is something that I will never forget. I heard on the radio that, when buying milk, you have to look at the bottom of the milk carton. There are numbers: 1, 2, 3, or 4. If the number 4 appears on the bottom, the milk was returned and sent back to the supermarket 4 times. So, everytime that I am going to buy milk, I buy milk that contains the number 1.

### Food-handler as a protagonist in the teaching-learning process

This thematic category assessed the food-handlers’ knowledge on the educational and training sessions. The guided questions were based on the training sessions and experiences and on the proposals for subsequent training sessions.

The food-handlers complained that the training sessions were repetitive, outdated, and non-innovative. Additionally, the material they learned could not be directly applied because of the lack of infrastructure in the schools.

This perspective was evident in the group meetings; the food-handlers noted the absence of innovation during the training sessions and the difficulty of applying in the school what they had learnt. Among the food-handlers, discontent was evident regarding the traditional training sessions where the food-handlers’ opinions are silenced by the professional trainers, their experiences are not shared, and the school's reality is not taken into account.

The focus group discussed their need to be heard during the training sessions and highlighted that the actual process is different from what they are taught and that the professional trainer should be aware of this:

The trainers throw all this information at you: ‘You have to do it!’; ‘That is the right way!’ Well, what might be right for you might not be right for me! Don't they want to know my opinion?…They are not living the school's reality so they do not know what is right! Sometimes, the trainers’ information might be applicable to other schools but not mine!

The group reinforced the importance for all food-handlers from a school to be simultaneously trained so that there is no difference in the information provided:

There should be training sessions for food-handlers in each of the schools. If there are 10 food-handlers in the school, all of them have to attend the training session.

The group also discussed the need to focus on relationships and humanization to minimize the social devaluation experienced by the food-handlers:

I think that food-handlers should be appreciated; it is an arduous job. I have been working here for the past 16 years preparing cold and hot meals, carrying light and heavy things…. My head is always overburdened by all the orders we receive.

## DISCUSSION

Some studies described the social feeding space as an interconnection between biological and cultural systems that contains different dimensions. One of these dimensions is the culinary space or kitchen where foods are transformed into a consumable product. This transformation takes place from technical operations, symbolic practices, and rituals (19,20). These concepts were found in the discussion of food-handlers who did not have the desired attitudes and practices but who reflected on their risk perception.

The food-handlers verbalized the knowledge they had acquired during training sessions, practical activities, and life experiences; however, they do not appropriately apply them in their daily activities. According to Slovic (11) and AAko (21), several factors influence risk perception, and the cultural component, which shows how risks are perceived, was highlighted. There are domestic risks which are perceived as voluntary, controllable, of low threat, and with natural origins. These domestic risks are more accepted than the involuntary risks which are unknown, life-threatening, and with human origins. For example, the individual considers that getting sick from inappropriate food-handling is considerably less risky to health than getting sick from a disease that was transmitted in an involuntary way.

Among the domestic risks, for example, the lack of hygienic practices during the food preparation might result in less aggressive health consequences because these are experienced on a daily basis. The involuntary risks are the ones with a high probability of harm, even if these are less frequent, that bring a feeling of fear and insecurity, such as being infected with the H1N1 virus or being involved in an airplane crash (11,21).

The risk perception influences the individual's behaviour and the level of precaution about situations that can cause accidents or lead to diseases. Cognitive and psychological aspects are related to this perception as demonstrated in studies that show the influence of social and cultural factors on the adoption of either prevention or risk behaviours (22,23). Clayton *et al*. studied the beliefs and practices of food-handlers regarding food safety and observed that, even though they knew what to do, 63% of the respondents admitted that they did not put their knowledge into practice. Furthermore, all the food-handlers had a perception that their work posed a low risk to the consumers’ health, even when the handlers prepared high-risk foods (14).

The low level of risk perception by the food-handlers was evident when food or silverware fell on the floor and were not properly disposed of or cleaned, suggesting that they are not aware of the risk to the students’ health. According to Slovic, these actions might be attributed to a lack of knowledge on the health consequences of this risk; a possible way to rectify this would be to make the food-handlers aware of the repercussions related with this risk (23).

A SAN training on the food acquisition and production should include not only the food-handlers but also the teachers, other staff, and the commensals (2,3).

The Brazilian legislation proposes the development of training sessions that focus on the shared responsibility relating to healthy and safe feeding and that involves students and the pedagogical team. The teacher needs to be a role model so the students can develop positive attitudes (24,25).

According to the food-handlers, the knowledge acquired during the training sessions reveals that the basic procedures, such as storage of prepared foods at low temperature and their re-use after appropriate heat treatment, are basically memorized. Costa, Lima, and Ribeiro who analyzed the training materials used in their study, identified that hygienic practices were emphasized during the training sessions performed by the professional trainers. Another study concluded that the training sessions provided information mostly on meal quality, hygienic practices during food production, and on schedules (5,26).

The information relating to the preparation of foods with unhealthy ingredients (i.e. sugar, salt, and fat) was not emphasized. National administration rule # 1010 about scholar feeding issues from the Health and Education Ministries (MS/MEC) proposes that the professionals involved in the production of school meals offer healthy foods to the students (2,27).

The focus group did not realize the importance of certain technical information, including the proper cleaning method for silverware, the relationship between storage temperature and bacterial growth, appropriate food-storage methods, and adequate meal transportation techniques. The most valued information by the food-handlers was obtained from sources that were questionable.

Food-handlers disregard important technical information and rely on memories from prior and current practices when performing their job functions. The information is then passed on to other food-handlers without the appropriate pedagogical techniques. On the other hand, this transfer of knowledge creates a bond among the food-handlers. Freire stated that individuals’ knowledge and understanding is not achieved by mechanical memorization but by the ability to understand the environment around them (9).

For this reason, Freire suggests that the students must be regarded as individuals and that the learning process should not be considered a simple transfer of information, which can lead to an absence of autonomy (9). He refers to the difficulty that educators encounter in finding language and syntax that the students can assimilate because the training does not present information that is close to the reality of the audience. It talks about “the utmost inattention to the harsh reality of the immense audience…” that, many times, is found in training sessions (28).

According to Freire, the mechanical memorization of the information and the inability to include the students in the learning process do not lead to individuals with critical and curious minds. This type of educator “cuts out the student's curiosity on behalf of the efficiency on the mechanical memorization of teaching, stopping the liberty of the student, the capacity of taking a chance; does not educate, it tames” (11).

Bellizi *et al*. performed a survey on the training sessions offered to food-handlers from different areas, with the objective of identifying the content of the training sessions, the pedagogical strategies, and difficulties encountered during the implementation of those training sessions. The lack of time, the absence of school superiors and of most food-handlers during the training sessions, and the alienation of the school administrative staff represent the biggest obstacles for an effective training session (10).

The importance of the training sessions and the implementation of different pedagogical techniques were the most important points in the risk perception of food safety and health by food-handlers (14,29).

The focus group noted a lack of interpersonal relationships and humanization during the training sessions. Some studies have revealed a level of discontent among the food-handlers with the accumulation of overlapping responsibilities, the absence of school activities, and the subservient relationship with the pedagogical team (4,30).

### Conclusions

This study assessed the aspects involved in the risk perception of food safety by food-handlers, a topic that has not been explored. The risk perception is a product of the individuals’ experiences, beliefs, and attitudes and not of their scientific knowledge; this study identified inappropriate procedures during the food preparation at schools. The most surprising findings were the low risk perception that food-handlers have (which can lead to serious health consequences) and the incorrect manner in which the food-handlers are trained. The food-handlers apply some of their knowledge on risk prevention based on the instruction of the professional trainer, not because they understand what constitutes a safe practice.

Thus, it is necessary to change the management of the production of school meals in a way that aims to reduce food safety risks, monitor hygienic practices during food production and train food-handlers in a constructive manner.

The risk perception of food safety is related to several members of the school community, which suggests that students, the pedagogical team, and the administrative staff should be encouraged to participate in activities concerning food safety. These measures will reduce risk, thereby leading to healthy and safe feeding practices at schools.

## ACKNOWLEDGEMENTS

We are grateful to the school food-handlers for their trust and their contribution to the study.
